# Comparative efficacy and safety of vaginal brachytherapy versus combined pelvic external beam radiotherapy and vaginal brachytherapy in managing intermediate to high-risk endometrial cancer: a systematic review and meta-analysis

**DOI:** 10.1186/s43046-025-00302-1

**Published:** 2025-06-03

**Authors:** Candra Novi Ricardo Sibarani, Siti Salima, Nicholas Adrianto

**Affiliations:** 1 Division of Gynecologic Oncology, Department of Obstetrics and Gynecology, Padjadjaran University, Dr. Hasan Sadikin General Hospital, Bandung, Indonesia; 2 School of Medicine and Health Sciences, Atma Jaya Catholic University of Indonesia, Pluit Raya No.2, Penjaringan, North Jakarta, Jakarta, Daerah Khusus Ibukota 14440 Indonesia

**Keywords:** Endometrial cancer, Meta-analysis, Radiation therapy, Brachytherapy

## Abstract

**Purpose:**

This review assesses the efficacy and safety of EBRT + VBT versus VBT alone in intermediate- to high-risk endometrial cancer.

**Methods:**

A systematic review and meta-analysis were conducted using PubMed, EMBASE, ProQuest, Ovid, and Scopus (until February 18, 2025). Studies comparing EBRT + VBT to VBT alone were included. The primary outcome was pelvic recurrence rate, while secondary outcomes included distant recurrence, overall survival, and toxicity. Data extraction, risk of bias assessment (RoB-2, ROBINS-I), and meta-analysis (random-effects models in RevMan) were performed. Certainty of evidence was evaluated using GRADE. PROSPERO registration: CRD420250654411.

**Results:**

Eight studies comprising 2,672 patients met inclusion criteria (1,347 received EBRT + VBT; 1,325 had VBT alone). EBRT + VBT significantly reduced pelvic recurrence (OR 0.14, *p* = 0.001) but showed no difference in vaginal recurrence (OR 0.25, *p* = 0.14), distant metastasis (OR 0.78, *p* = 0.45) or overall survival (HR 0.82, *p* = 0.29, I^2^ = 72%). EBRT + VBT was associated with higher gastrointestinal, genitourinary, and hematologic toxicity.

**Conclusion:**

EBRT + VBT improves pelvic control but does not enhance survival and increases toxicity. VBT alone remains a viable option, highlighting the need for individualized treatment strategies.

## Introduction

Endometrial cancer is the most prevalent gynecologic malignancy in developed countries, with over 400,000 new cases diagnosed annually worldwide [1]. While early-stage disease is associated with a favorable prognosis, intermediate- to high-risk cases comprise 20–30% of diagnoses and carry an increased risk of pelvic recurrence and distant metastasis [2, 3]. Postoperative adjuvant treatment for early-stage endometrial cancer is tailored based on individual patient risk factors [4]. There is considerable variability in therapeutic strategies employed across different countries and even among institutions within the same country [5]. Currently, surgery followed by observation is deemed appropriate for patients with early endometrial cancer exhibiting low-risk pathological features [6]. In contrast, adjuvant radiation therapy is recommended for those identified as having an intermediate to high risk of recurrence, supported by several randomized trials that have demonstrated its efficacy in improving outcomes [6, 7]. Adjuvant radiotherapy remains a fundamental component of postoperative management in this population; however, the optimal radiation approach—external beam radiotherapy (EBRT) alone versus combined EBRT and vaginal brachytherapy (VBT)—remains a subject of ongoing investigation and debate [8, 9].

Current guidelines recommend EBRT for pelvic control in high-risk patients [10]. Vaginal brachytherapy has been shown to offer advantages in achieving better quality of life and favorable outcomes, including improvements in global health status as well as physical and social functioning. From an economic standpoint, vaginal brachytherapy has been reported to be cost-effective compared to observation for patients with high-intermediate-risk or intermediate-risk diseases [5]. Furthermore, the use of external beam radiation therapy, whether alone or in combination with vaginal brachytherapy, has not demonstrated significant improvements in survival outcomes. However, emerging evidence suggests that the addition of VBT to EBRT may enhance vaginal cuff control, a common site of recurrence, while potentially reducing toxicity compared to extended-field EBRT [6]. Conversely, critics argue that the incremental benefit of VBT may be marginal in patients already receiving pelvic EBRT, with concerns about overtreatment and increased morbidity [11].

This controversy is reflected in heterogeneous clinical practices globally, with some centers favoring combined therapy and others relying on EBRT alone. Despite randomized trials such as PORTEC-2 establishing the non-inferiority of VBT alone compared to EBRT in intermediate-risk cohorts, direct comparisons of EBRT + VBT versus EBRT alone in intermediate- to high-risk patients are limited to small retrospective studies and underpowered trials [7]. A synthesis of existing data is urgently needed to clarify whether the addition of VBT provides meaningful clinical benefits that outweigh its risks in this population. This review aims to evaluate and compare the efficacy and safety profiles of EBRT combined with VBT against VBT alone in patients diagnosed with intermediate to high-risk endometrial cancer.

## Methods

### Search strategy and

A comprehensive literature search was performed across multiple databases, including Pubmed, Cochrane Central Register of Controlled Trials (CENTRAL), Google Scholar, Ovid, ProQuest, and Scopus from their inception until February 18, 2025. The search strategy utilized a combination of controlled vocabulary and keywords related to endometrial cancer, EBRT, and VBT. No language restrictions were applied, and studies published only as abstracts were included. Additionally, reference lists of relevant articles were reviewed to identify further studies.

### Inclusion criteria

Studies were included if they met the following criteria. Women with intermediate to high-risk endometrial cancer, defined by risk factors such as grade 3 tumors, more than 50% myometrial invasion, lymphovascular space invasion (LVSI), or advanced stage I–III. Studies comparing EBRT ± VBT versus VBT alone or no radiotherapy. Retrospective or prospective cohort studies, randomized controlled trials (RCTs), or large database analyses. Published in English or with English translations. Studies were excluded from this review based on specific criteria. Those that focused exclusively on low-risk patients or stage II cases without appropriate risk stratification were not considered. Additionally, case reports, reviews, and non-comparative studies were also excluded, ensuring that only relevant comparative research was included in the analysis. Our primary outcome was the pelvic recurrence rate. Secondary outcomes included distant recurrence rate, overall survival, toxicity rate.

### Data extraction and risk of bias assessment

Our study followed the reporting standards outlined in the Preferred Reporting Items for Systematic Reviews and Meta-Analyses (PRISMA) guidelines [12]. Two independent reviewers screened abstracts and full texts for eligibility. Data were extracted using a standardized form, and the risk of bias was assessed using the Version 2 of the Cochrane risk-of-bias tool for randomized trials (RoB-2) and Risk of Bias in Non-randomized Studies of Interventions (ROBINS-I) tool [13, 14]. Disagreements were resolved through consensus.

### Statistical analysis

Meta-analysis was performed using Review Manager software (RevMan) [15]. Random effects models were employed to synthesize quantitative data and calculate odds ratios (ORs) with 95% confidence intervals (CIs). Heterogeneity among studies was assessed using the I^2^ statistic, with I^2^ > 50% indicating substantial heterogeneity. The certainty of evidence was evaluated using the GRADE (Grading of Recommendations, Assessment, Development, and Evaluation) framework, in accordance with established methodology. The assessment was conducted utilizing the GRADEpro Guideline Development Tool (McMaster University, 2015), developed by Evidence Prime Inc., and accessible via GRADEpro.org [16]. This systematic review and meta-analysis has been registered on PROSPERO under the registration number CRD420250654411.

## Results

**Fig. 1:**
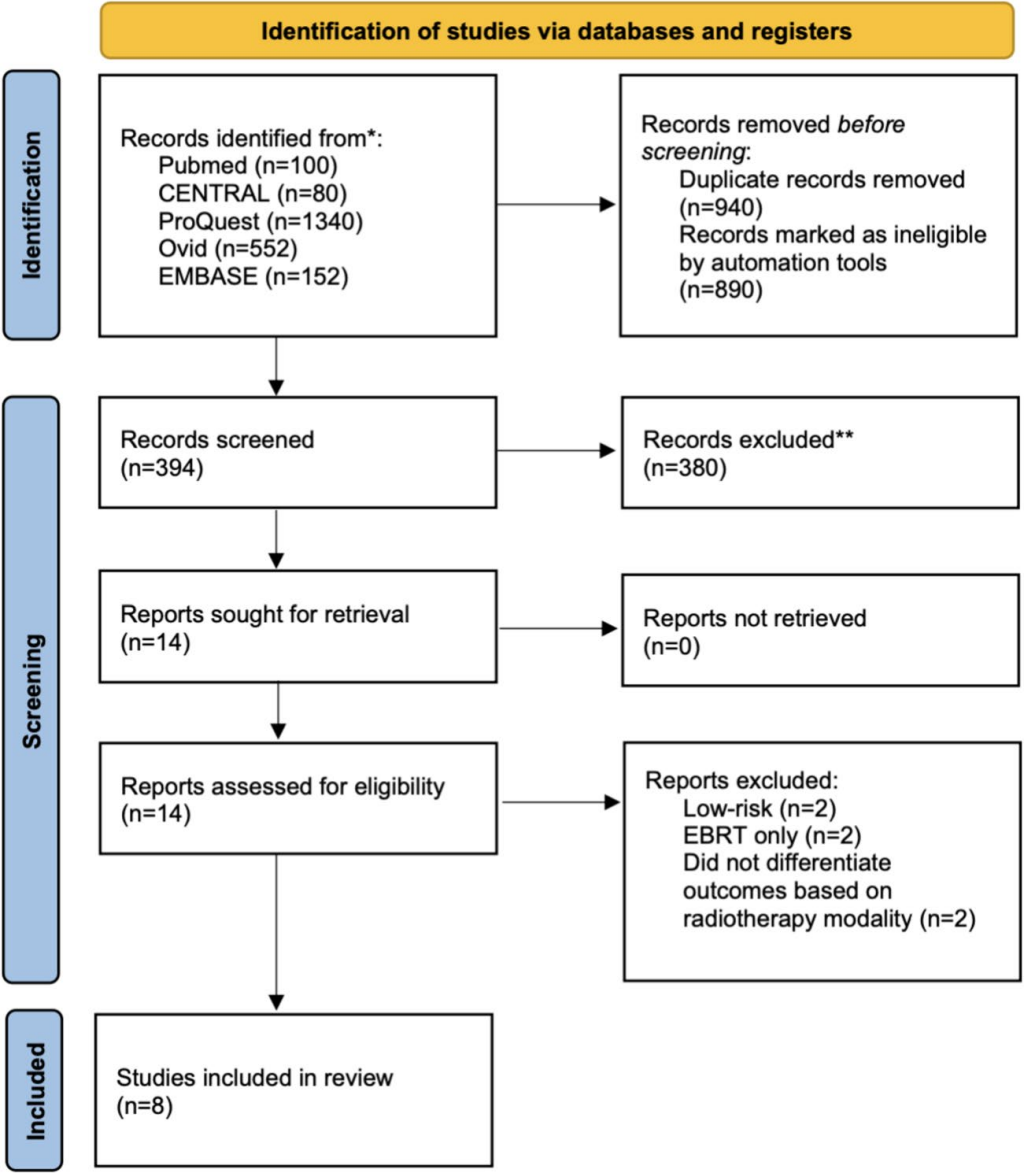
The Preferred Reporting Items for Systematic Reviews and Meta-Analysis flow diagram eligible for this meta-analysis. The diagram summarizes the search strategy and selection process to include articles [12]

A total of eight studies met the inclusion criteria (Fig. 1) and analyzed 2,672 patients [5, 11, 17–22]. Among them, 1,347 patients (50.4%) received EBRT ± VBT, while 1,325 patients (49.6%) underwent VBT alone. The study characteristics are summarized in Table 1.The study populations were heterogeneous, with a mean age ranging from 54 to 69. The predominant histological subtype was endometrioid carcinoma, and the majority of patients were classified as FIGO stage I to II.

**Table 1:** Characteristics of studies included

Hass P et al., 2018 [20]			Sunil RA et al., 2018 [11]		Sorbe B et al., 2012 [17]		Jin M et al., 2019 [5]		Chitapanarux N et al., 2024 [18]		Hou X-R et al., 2019 [21]		Ren K et al., 2021 [28]		Wang et al., 2021 [22]	
EBRT ± VBT (*n* = 418)		VBT (*n* = 430)	EBRT + VBT (*n* = 25)	VBT (*n* = 25)	EBRT + VBT (*n* = 264)	VBT (*n* = 263)	VBT + EBRT (*n* = 119)	VBT (*n* = 119)	EBRT + VBT (*n* = 80)	VBT (*n* = 65)	EBRT + VBT (*n* = 145)	VBT (*n* = 245)	EBRT + VBT (*n* = 154)	VBT (*n* = 153)	EBRT + VBT (*n* = 142)	VBT (*n* = 25)
Intermediate risk (*n* = 311) High risk (*n* = 436)			Intermediate and high-risk		Medium risk		Intermediate-high risk		High risk		Intermediate-high risk		HIR: 49 High-risk: 105	HIR: 115 High-risk: 38	High-risk	
Study Design	Retrospective study		Randomized controlled trial		Randomized controlled trial		Retrospective study		Retrospective study		Retrospective study		Retrospective study		Retrospective study	
Country	Germany		India		Sweden		China		Thailand		China		China		China	
Age (years)	69 (34–89)	69 (31–96)	57.40 ± 6.6	54.96 ± 8.3	68	68	58 (33–76)	58 (30–85)	59 (54–65.5)	58 (54–63)	58 (28–85)		< 60: 209 > 60: 98		54.36 (34–75)	60.96 (42–85)
Histological type																
Endometrioid	335 (80.1)	386 (89.8)	-	-	-	-	-	-	64 (80)	65 (100)	-	-	-	-	154	25
Non- endometrioid	31 (7.4)	10 (2.3)	-	-	-	-	-	-	14 (17.5)	-	-	-	-	-	-	-
Others	52 (12.4)	34 (7.9)	-	-	-	-	-	-	2 (2.5)	-	-	-	-	-	-	-
Type I	-	-	-	-	-	-	-	-	-	-	-	-	262		-	-
Type II	-	-	-	-	-	-	-	-	-	-	-	-	45		-	-
FIGO Stage																
Stage I	390 (39.5)	239 (64.6)	-	-	-	-	-	-	-	-	-	-	-	-	-	-
Stage IA	-	-	8 (32.0)	12 (48.0)	26 (9.9)	18 (6.8)	39 (32.8)	39 (32.8)	10 (12.5)	35 (53.8)	183 (46.9)		143		-	-
Stage IB	-	-	17 (68.0)	13 (52.0)	99 (37.5)	100 (38)	77 (64.7)	77 (64.7)	31 (38.8)	30 (46.2)	207 (53.1)		102		44 (31)	20 (80)
Stage IC	-	-	-	-	139 (52.7)	145 (55.1)	-	-	-	-	-	-	-		-	-
Stage II	76 (20.5)	42 (6.5)	-	-	-	-	3 (2.5)	3 (2.5)	-	-	-	-	62		98 (69)	5 (20)
Stage IIA	-	-	-	-	-	-	-	-	18 (22.5)	-	-	-	-	-	-	-
Stage IIB	-	-	-	-	-	-	-	-	-	-	-	-	-	-	-	-
Stage III	54 (14.6)	49 (7.6)	-	-	-	-	-	-	-	-	-	-	-	-	-	-
Stage IIIA	-	-	-	-	-	-	-	-	10 (12.5)	-	-	-	-	-	-	-
Stage IIIB	-	-	-	-	-	-	-	-	1 (1.3)	-	-	-	-	-	-	-
Stage IIIC1	-	-	-	-	-	-	-	-	7 (8.8)	-	-	-	-	-	-	-
Stage IIIC2	-	-	-	-	-	-	-	-	2 (2.5)	-	-	-	-	-	-	-
Stage IV	1 (0.3)	5 (0.8)	-	-	-	-	-	-	-	-	-	-	-	-	-	-
Histological grade																
1	126 (30.9)	71 (18.5)	6 (24.0)	7 (28.0)	-	-	37 (31.1)	41 (34.5)	23 (28.8)	23 (35.4)	100 (25.6)		-	-	32 (25.5)	3 (12)
2	209 (51.2)	182 (47.5)	13 (52.0)	15 (60.0)	-	-	39 (32.8)	43 (36.1)	16 (20)	20 (30.8)	201 (51.5)		-	-	47 (33.1)	2 (8)
3	73 (17.9)	130 (33.9)	6 (24.0)	3 (12.0)	-	-	43 (36.1)	33 (29.4)	36 (45)	22 (33.8)	89 (22.8)		-	-	63 (44.4)	20 (80)
NA	-	-	-	-	-	-	-	-	5 (6.3)	-	-	-	-	-	-	-

*EBRT* External beam radiotherapy, *VBT* Vaginal brachytherapy, *FIGO* The International Federation of Gynecology and Obstetrics, *NA* Not available

### Recurrence

**Fig. 2:**
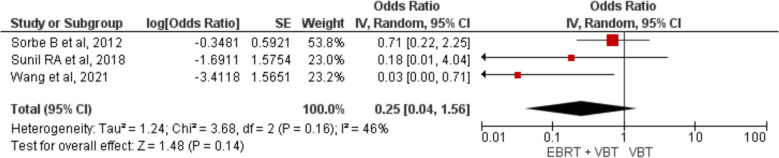
Vaginal recurrence

The pooled analysis from two studies revealed an OR of 0.25 (95% CI; 0.04–1.56) for EBRT + VBT compared to VBT, indicating no significant difference in odds of events between the two groups (Fig. 2, Table 2) [11, 17]. There was no heterogeneity among the studies (Chi^2^ = 3.68, *p* = 0.16, I^2^ = 46%), confirming consistent results. The overall effect test yielded a Z statistic of 1.48 (*p* = 0.14).

**Table 2:** Recurrence

**No**	**Author**		**Recurrence rate (%)**	***p*-value**	**Vaginal recurrence (%)**	***p*-value**	**Pelvic recurrence (%)**	***p*-value**	**Distant Metastasis (%)**	***p*-value**
1	Hass P et al., 2018 [20]	EBRT ± VBT (*n* = 418)	-	-	-	-	-	-	-	-
		VBT (*n* = 430)	-		-		-		-	
2	Sunil RA et al., 2018 [11]	EBRT + VBT (*n* = 25)	-	-	0/25 (0)	0.15	0/25 (0)	-	1/25 (4)	-
		VBT (*n* = 25)	-		2/25 (8)		2/25 (8)		3 (12)	
3	Sorbe B et al., 2012 [17]	VBT + EBRT (*n* = 264)	15 (5.7)	0.052	5 (1.9)	0.555	1 (0.4)	0.0006	12 (4.6)	0.334
		VBT (*n* = 263)	27 (10.3)		7 (2.7)		14 (5.3)		17 (6.5)	
4	Jin M et al., 2019 [5]	VBT + EBRT (*n* = 119)	-	-	-	-	-	-	8 (6.7)	1.0
		VBT (*n* = 119)	-	-	-	-	-	-	8 (6.7)	
5	Chitapanarux N et al., 2024 [18]	VBT + EBRT (*n* = 80)	-	-	-	-	-	-	-	-
		VBT (*n* = 65)	-	-	-	-	-	-	-	-
6	Hou X-R et al., 2019 [21]	EBRT + VBT (*n* = 145)	22 (5.6)	-	4 (1)	-	7 (1.8)	-	19 (4.9)	-
		VBT (*n* = 245)								
7	Ren K et al., 2021 [28]	EBRT + VBT (*n* = 154)							20 (13.13)	0.30
		VBT (*n* = 153)							12 (8.02)	
8	Wang et al., 2021 [22]	EBRT + VBT (*n* = 142)	-	-	0 (0)	-	4 (2.8)	-	13 (9.2)	-
		VBT (*n* = 25)	-		2 (8)		3 (12)		6 (24)	

*EBRT* External beam radiotherapy, *VBT* Vaginal brachytherapy

**Fig. 3:**
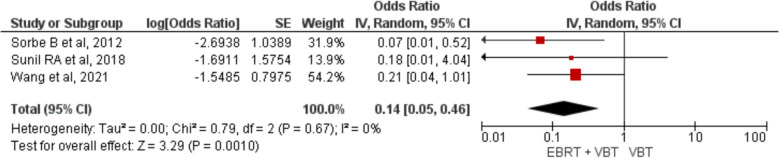
Pelvic recurrence

The pooled analysis from two studies showed an OR of 0.14 (95% CI;0.05–0.46) for EBRT + VBT compared to VBT, indicating significantly lower odds of events in the EBRT + VBT group (Fig. 3, Table 2) [11, 17]. There was no heterogeneity among the studies (Chi^2^ = 0.79, *p* = 0.67, I^2^ = 0%), confirming consistent results. The overall effect test yielded a Z statistic of 3.29 (*p* = 0.0010).

**Fig. 4:**
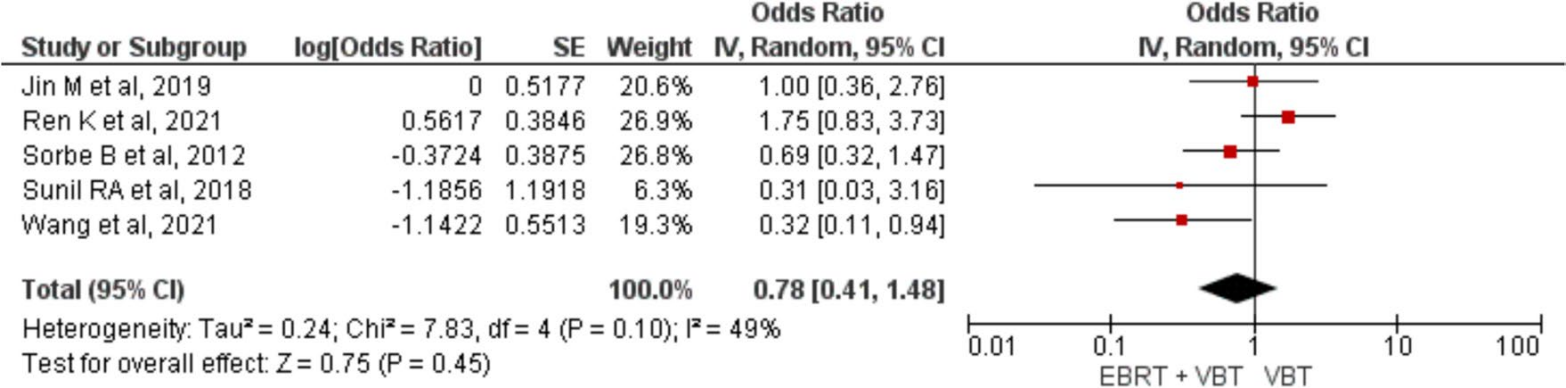
Distant metastasis

The pooled analysis revealed an OR of 0.78 (95% CI; 0.41–1.48) for EBRT + VBT compared to VBT, indicating no significant difference in odds of events between the two groups (Fig. 4, Table 2). There was no heterogeneity among the studies (Chi^2^ = 7.83, *p* = 0.10, I^2^ = 49%), confirming consistent results. The overall effect test yielded a Z statistic of 0.75 (*p* = 0.45).

### Survival

**Fig. 5:**
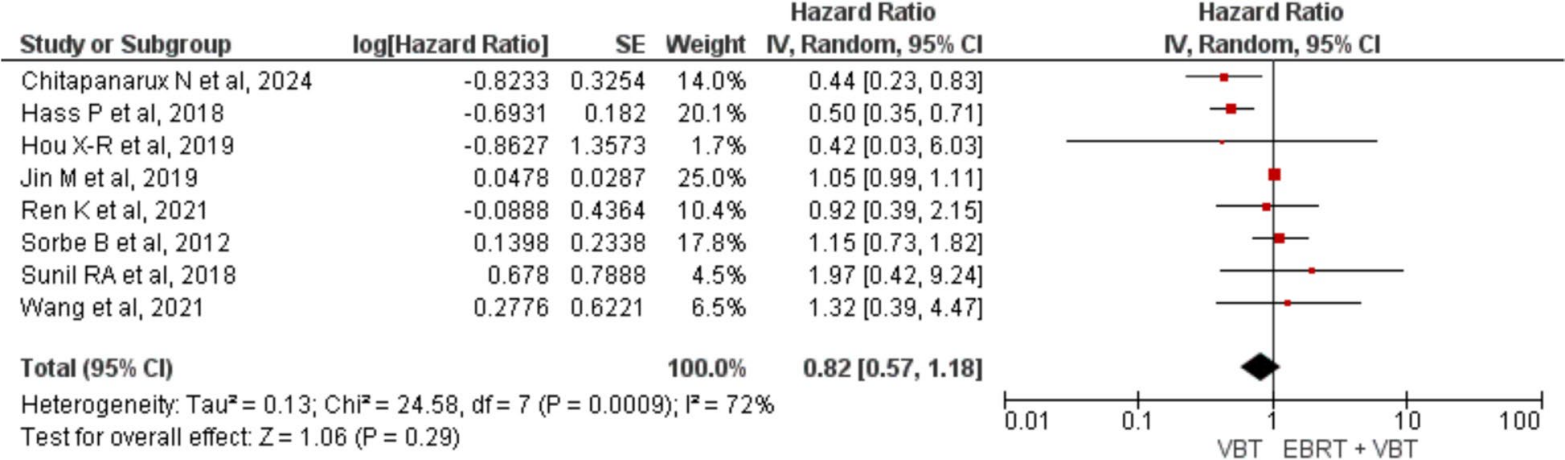
Overall survival

The pooled analysis showed no statistically significant difference in the risk of events (e.g., recurrence or mortality) between VBT and EBRT + VBT, with an overall HR of 0.82 (95% CI;0.57–1.18) (Fig. 5, Table 3). Substantial heterogeneity was observed (Tau2 = 0.13, Chi2 = 24.58, *p* = 0.0009, I2 = 72%). However, the overall effect test (Z = 1.06, *p* = 0.29) confirmed no significant difference in OS. The heterogeneity observed in the pooled analysis of studies comparing VBT and EBRT + VBT may be due to several factors. These include differences in study designs, such as randomized controlled trials versus retrospective analyses, which can lead to variations in outcomes. Additionally, patient characteristics, such as age, disease stage, and comorbidities, may differ across studies, affecting treatment responses. Variations in treatment protocols, dosages, and follow-up durations can also contribute to the observed heterogeneity.

**Table 3:** Survival

**No**	**Author**		**OS**	**HR**	**PFS**	**HR**	**RFS**	**HR**	**DFS**	**HR**	**LRFS**	***p*-value**	**RRFS**	***p*-value**	**DMFS**	***p*-value**
1	Hass P et al., 2018 [20]	EBRT ± VBT (*n* = 418)	259 (62)	1.00	-	-	-	-	-	-	-	-	-	-	-	-
		VBT (*n* = 430)	345 (80.2)	0.50 (0.35–0.72) (*p* < 0.0001)	-	-	-	-	-	-	-	-	-	-	-	-
2	Sunil RA et al., 2018 [11]	EBRT + VBT (*n* = 25)	23 (92) (72.0–98.0)	1.00	-	-	-	-	96% (77.0–99.0)	2.30 (0.23–23.05) *p* = 0.30	-	-	-	-	-	-
		VBT (*n* = 25)	21 (84) (63.0–95.0)	1.97 (0.36–10.86) *p* = 0.39	-	-	-	-	88% (68.0–97.0)		-	-	-	-	-	
3	Sorbe B et al., 2012 [17]	VBT + EBRT (*n* = 264)	235 (88.9)	1.00	-	-	86.7%	1.17 (0.77–1.77) *p* = 0.46	-	-	-	-	-	-	-	-
		VBT (*n* = 263)	233 (88.8)	1.15 (0.73–1.80) (*p* = 0.55)	-	-	86.2%		-	-	-		-	-	-	-
4	Jin M et al., 2019 [5]	VBT + EBRT (*n* = 119)	112 (93.9) (95%CI; 86.8–97.2)	1.00	90% (95%CI; 82.2–94.6)	2.309 (1.074–4.962) *p* = 0.032	-	-	-	-	-	-	-	-	-	-
		VBT (*n* = 119)	113 (94.8) (95%CI; 87.8–97.8)	1.049 (0.992–1.110) *p* = 0.095	84.4% (95%CI; 74.9–90.5)		-	-	-	-	-		-	-	-	-
5	Chitapanarux N et al., 2024 [18]	VBT + EBRT (*n* = 80)	52 (65)	1.00	-	-	-	-	-	-	95.6%	-	99.1%	-	92.5%	-
		VBT (*n* = 65)	55 (84.4)	0.439 (0.232–0.833) *p* = 0.021	-	-	-	-	-	-	93.3%		100.0%		100.0%	-
6	Hou X-R et al., 2019 [21]	EBRT + VBT (*n* = 145)	5 year: 137 (94.7) 10 year: 128 (88.4)	1.00	-	-	-	-	-	-	-	-	-	-	-	-
		VBT (*n* = 245)	5 year: 240 (97.8) 10 year: 231 (94.2)	0.422 (0.131–1.363) *p* = 0.525	-	-	-	-	-	-	-	-	-	-	-	-
7	Ren K et al., 2021 [28]	EBRT + VBT (*n* = 154)	143 (92.6) (95% CI 88%–­ 96.4%)	1.00	-	-	-	-	-	-	-	-	-	-	-	-
		VBT (*n* = 153)	143 (93.2) (95% CI 88.9%–97.5%)	0.915 (0.389–2.152) *p* = 0.838	-	-	-	-	-	-	-	-	-	-	-	-
8	Wang et al., 2021 [22]	EBRT + VBT (*n* = 142)	3 year OS: 135/142 (95.1)	-	-	-	-	-	130/142 (91.2)	-	134/142 (94.4)	-	-	-	-	-
		VBT (*n* = 25)	3 year OS: 21 (85.2)	1.32 (0.39–4.46) *p* = 0.66	-		-		18/25 (72.4)		19/25 (76.5)		-		-	

*OS* Overall survival, *HR* Hazard ratio, *PFS* Progression-free survival, *DFS* Disease-free survival, *DM* Distant metastasis, *LRFS* Local Recurrence-Free Survival, *RRFS* Regional Recurrence-Free Survival, *DMFS* Distant Metastasis-Free Survival

Several studies evaluated local recurrence-free survival (LRFS), regional recurrence-free survival (RRFS), distant metastasis-free survival (DMFS), recurrence-free survival (RFS), and disease-free survival (DFS), yielding mixed results. Chitapanarux et al. reported slightly higher LRFS and RRFS in the VBT-alone group than EBRT + VBT, suggesting that VBT alone may provide sufficient local and regional control without additional EBRT [18]. Findings on DMFS were inconsistent, with Chitapanarux et al. favoring VBT alone [18]. In contrast, Jin et al. and Sorbe et al. found no significant difference, indicating that EBRT may not improve distant metastasis outcomes. Similarly, RFS results varied, with Jin et al. reporting a significant improvement with VBT, while Sorbe et al. observed no difference between treatment groups, questioning the added benefit of EBRT [5, 17]. Likewise, Wang et al. found no significant difference in DFS between VBT alone and EBRT + VBT, reinforcing the notion that VBT alone may be adequate for disease control in select patients [22].

### Safety

**Fig. 6:**
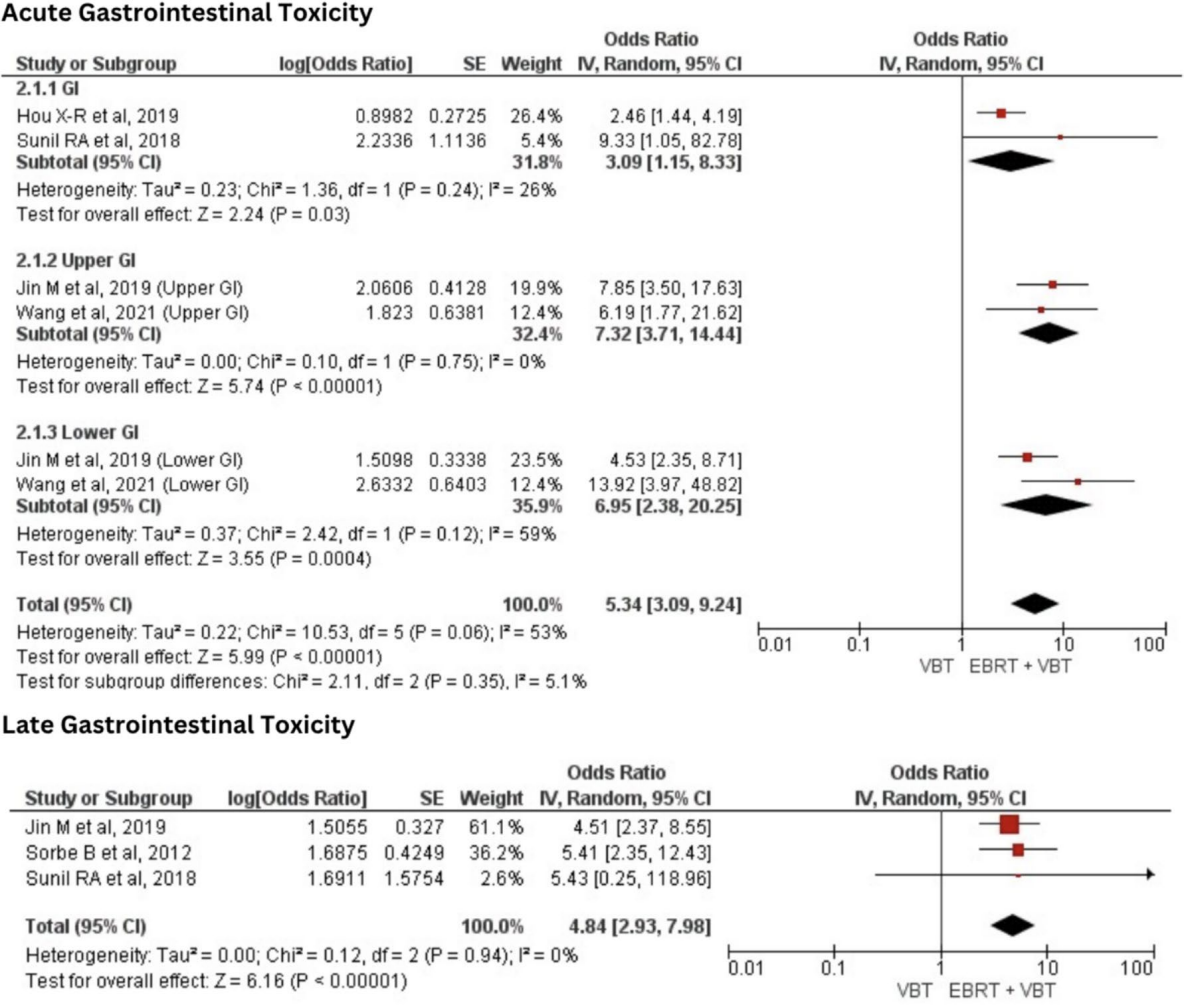
Acute gastrointestinal toxicity (top). Late gastrointestinal toxicity (bottom)

The pooled analysis showed a significantly higher risk of acute GI toxicity in patients receiving EBRT + VBT compared to VBT alone (OR 5.34, 95% CI; 3.09–9.24, I2 = 53%) (Fig. 6, Table 4). Subgroup analysis revealed elevated toxicity in both upper GI (OR: 7.32, 95% CI; 3.71–14.44) and lower GI (OR 6.95, 95% CI: 2.38–20.25, *P* = 0.0004), with moderate heterogeneity (I2 = 53%). Subgroup differences were not statistically significant (Chi2 = 2.11, *P* = 0.35, I2 = 5.1%), indicating a consistent increased risk across GI regions. For late GI toxicity, EBRT + VBT also showed a significantly increased risk (OR 4.84, 95% CI; 2.93–7.98) with no significant heterogeneity (I2 = 0%).

**Table 4:** Safety

	Hass P et al., 2018 [20]		Sunil RA et al., 2018 [11]		Sorbe B et al., 2012 [17]		Jin M et al., 2019 [5]		Chitapanarux N et al., 2024 [18]		Hou X-R et al., 2019 [21]		Wang et al., 2021 [22]	
	EBRT ± VBT (*n* = 418)	VBT (*n* = 430)	EBRT + VBT (*n* = 25)	VBT (*n* = 25)	VBT + EBRT (*n* = 264)	VBT (*n* = 263)	VBT + EBRT (*n* = 119)	VBT (*n* = 119)	VBT + EBRT (*n* = 80)	VBT (*n* = 65)	EBRT + VBT (*n* = 145)	VBT (*n* = 245)	EBRT + VBT (*n* = 142)	VBT (*n* = 25)
**Acute**														
GI (%)	-	-	7 (30)	1 (2)	-	-	Upper GI: Grade 1–2: 43 (36.1) Lower GI: Grade 1–2: 47 (39.5)	Upper GI Grade 1–2: 8 (6.7) Lower GI: Grade 1–2: 15 (12.6)	-	-	Grade 1–2: 37/145 (22.2)	Grade 1–2: 30/245 (12.2)	**Upper GI:** Grade 1–2: 65 (45.8) Grade 3: 0 **Lower GI:** Grade 1–2: 93 (65.5) Grade 3: 1 (0.7)	**Upper GI:** Grade 1–2: 3 (12.0) Grade 3: 1 (4.0) **Lower GI:** Grade 1–2: 3 (12.0) Grade 3: 0 (0)
Genitourinary	-	-	Low grade 7 (30) High grade: 3 (12)	Low grade 1 (2) High grade: 0%	-	-	Grade 1–2: 37 (31.1)	Grade 1–2: 8 (6.7)	-	-	44 (11.3)		Grade 1–2: 18 (12.7) Grade 3: 1 (0.7)	Grade 1–2: 2 (8.0)
Hematological	-	-	-	-	-	-	Grade 1–2: 17 (14.3)	Grade 1–2: 10 (8.4)	-	-	-	-	Grade 1–2: 60 (42.3) Grade 3: 7 (4.9)	Grade 1–2: 4 (16.0)
Anemia	-	-	-	-	-	-	-	-	Grade 0–2: 77 (96.3) Grade 3–4: 2 (2.5) N/A: 1 (1.3)	Grade 0–2: 65 (100)	-	-		
Neutropenia	-	-	-	-	-	-	-	-	Grade 0–2: 78 (97.5) Grade 3–4: 2 (2.5)	Grade 0–2: 65 (100)	-	-		
Thrombocytopenia	-	-	-	-	-	-	-	-	Grade 0–2: 78 (97.6) Grade 3–4: 1 (1.3) N/A: 1 (1.3)	Grade 0–2: 65 (100)	-	-		
Dermatitis/Skin	-	-	Low grade: 6 (24) High grade: 14%	Low grade 5 (20) High grade: 0%	-	-	-	-	Grade 0–2: 79 (98.8) N/A: 1 (1.3)	Grade 0–2: 65 (100)	-	-		
Diarrhea	-	-	-	-	-	-	-	-	Grade 0–2: 74 (92.6) Grade 3–4: 6 (7.4)	Grade 0–2: 64 (98.4) N/A: 1 (1.6)	-	-		
Cystitis	-	-	-	-	-	-	-	-	Grade 0–2: 79 (98.8) N/A: 1 (1.3)	Grade 0–2: 65 (100)	-	-		
**Late**														
GI	-	-	2 (6)	0 (0)	Grade 1: 26 (9.8) Grade 2: 8 (2.9) Grade 3: 4 (1.8)	Grade 1: 6 (2.3) Grade 2: 1 (0.4) Grade 3: 0 (0)	Grade 1–2: 49 (41.2)	Grade 1–2: 16 (13.4)	-	-	Grade 3: 2 (0.1)	0 (0)	-	-
Genitourinary	-	-	-	-	Grade 1: 71 (26.9) Grade 2: 17 (6.4) Grade 3: 5 (1.9)	Grade 1: 53 (20.2) Grade 2: 7 (2.7) Grade 3: 2 (0.8)	Grade 1–2: 16 (3.4)	Grade 1–2: 6 (5.0)	-	-	-	-	-	-
Hematological	-	-	-	-	-	-	Grade 1–2: 20 (16.8)	Grade 1–2: 9 (7.6)	-	-	-	-	-	-
Vagina	-	-	-	-	Grade 1: 34 (12.7) Grade 2: 2 (0.7) Grade 3: 0 (0)	Grade 1: 11 (4.1) Grade 2: 55 (20.8) Grade 3: 2 (0.8)	-	-	-	-	-	-	-	-

*EBRT* External beam radiotherapy, *VBT* Vaginal brachytherapy, *GI* Gastrointestinal

**Fig. 7:**
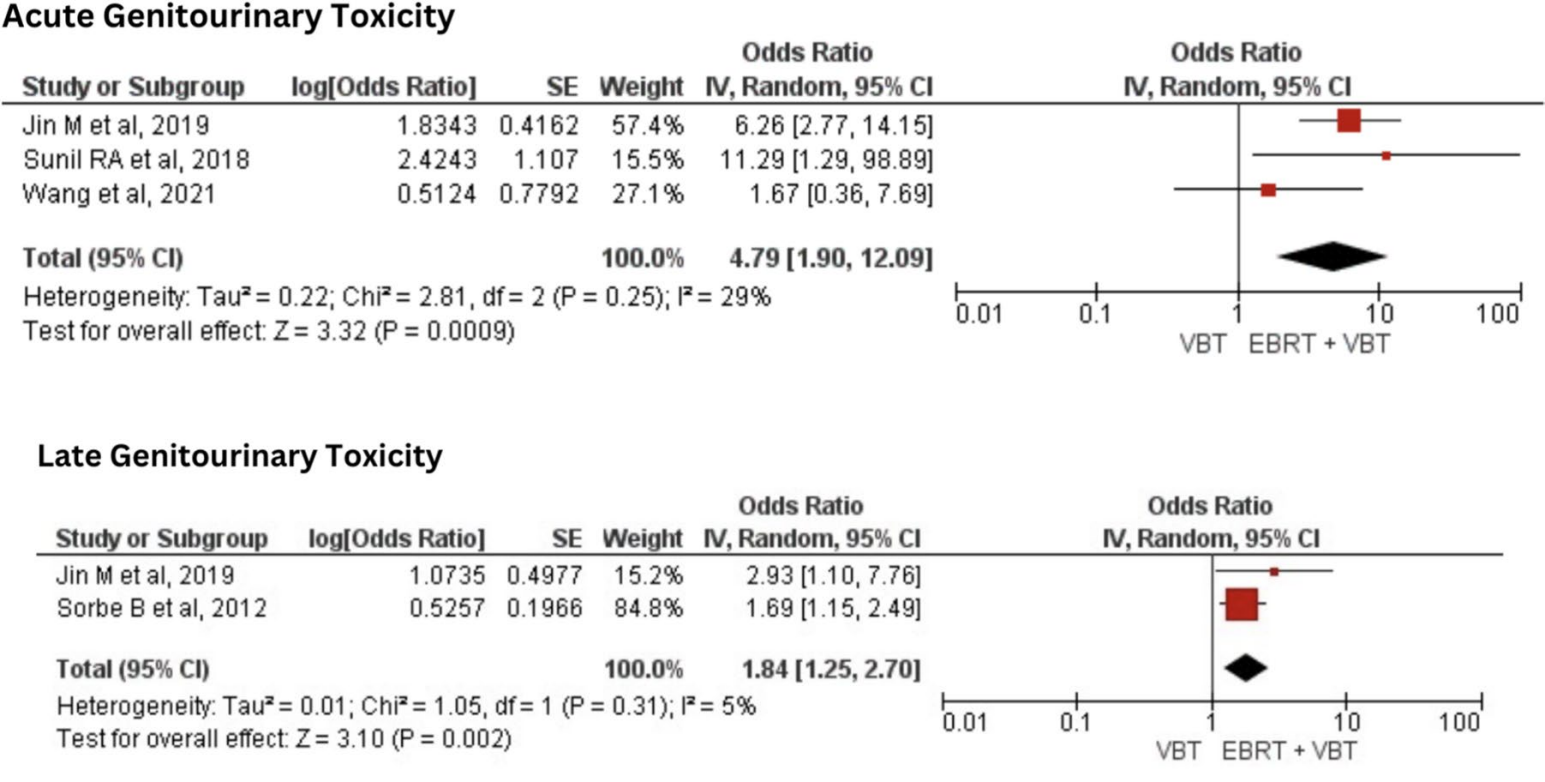
Acute genitourinary toxicity (top) and late genitourinary toxicity (bottom)

The pooled analysis for acute genitourinary toxicity showed an odds ratio (OR) of 6.74 (95% CI; 3.14–14.45) for EBRT + VBT compared to VBT (Fig. 7, Table 4). There was no heterogeneity among the studies (Chi2 = 0.25, *p* = 0.62, I2 = 0%). The overall effect test yielded a Z statistic of 4.90 (*p* < 0.00001). For late genitourinary toxicity, the pooled analysis showed an OR of 1.84 (95% CI; 1.25–2.70), with minimal heterogeneity (Chi2 = 1.05, *p* = 0.31, I2 = 5%). The overall effect test yielded a Z statistic of 3.10 (*p* = 0.002).

**Fig. 8:**
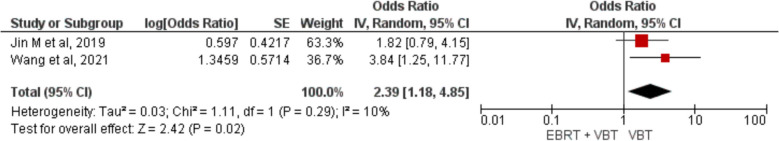
Hematological toxicity

The pooled analysis for acute hematological toxicity showed an OR of 2.39 (95% CI; 1.18–4.85) for EBRT + VBT compared to VBT (Fig. 8, Table 4). There was no heterogeneity among the studies (Chi2 = 1.11, *p* = 0.29, I2 = 10%). The overall effect test yielded a Z statistic of 2.42 (*p* = 0.002).

**Fig. 9:**
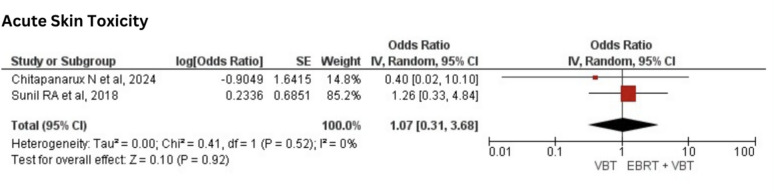
Acute skin toxicity

The pooled analysis for acute skin toxicity showed an OR of 1.07 (95% CI; 0.31–3.68) for EBRT + VBT compared to VBT (Fig. 9, Table 4). There was no heterogeneity among the studies (Chi2 = 0.41, *p* = 0.52, I2 = 0%). The overall effect test yielded a Z statistic of 0.10 (*p* = 0.92).

### Confidence in cumulative evidence

The studies included in this meta-analysis comprised RCTs and cohort studies, initially indicating high-quality evidence. However, the overall certainty varied due to methodological limitations. Evidence for vaginal and pelvic recurrence was rated as moderate, primarily due to imprecision, though sensitivity analyses suggested minimal bias impact. In contrast, distant metastasis and overall survival were rated as very low due to serious concerns regarding risk of bias, inconsistency, and imprecision, with study design variability and wide confidence intervals contributing to downgrades. Toxicity outcomes showed mixed certainty, with acute and late gastrointestinal toxicity, acute genitourinary toxicity, and lower gastrointestinal toxicity rated as moderate due to retrospective study designs and potential selection bias, while hematological toxicity was rated as low due to inconsistencies and differences in patient management. Moderate-to-high inconsistencies were observed, likely due to treatment protocol variations and population differences, and publication bias could not be assessed due to the limited number of studies. Overall, the evidence ranged from low to moderate certainty, with some outcomes downgraded to very low due to methodological concerns, as summarized in Table 5.

**Table 5:** GRADE evidence profile

Certainty assessment							№ of patients		Effect		Certainty
**№ of studies**	**Study design**	**Risk of bias**	**Inconsistency**	**Indirectness**	**Imprecision**	**Other considerations**	**VBT**	**EBRT vs VBT**	**Relative** **(95% CI)**	**Absolute** **(95% CI)**	
**Vaginal Recurrence**											
3	2 RCTs + 1 Cohort	not serious	not serious^a^	not serious	serious^b^	none	11/313 (3.5%)	5/431 (1.2%)	**OR 0.25** (0.04 to 1.56)	**1 fewer per 100** (from 1 fewer to 1 more)	⨁⨁⨁◯ Moderate^a,b^
**Pelvic Recurrence**											
3	2 RCTs + 1 Cohort	not serious	not serious	not serious	serious^b^	none	19/313 (6.1%)	5/431 (1.2%)	**OR 0.14** (0.05 to 0.46)	**1 fewer per 100** (from 1 to 1 fewer)	⨁⨁⨁◯ Moderate^b^
**Distant Metastasis**											
5	2 RCTs + 3 Cohort	serious^c^	serious^d^	not serious	serious^e^	none	46/585 (7.9%)	54/704 (7.7%)	**OR 0.78** (0.41 to 1.48)	**2 fewer per 100** (from 4 fewer to 3 more)	⨁◯◯◯ Very low^c,d,e^
**Overall Survival**											
8	2 RCTs + 6 Cohort	serious^f^	serious^g^	not serious	serious^h^	none	154/1325 (11.6%)	251/1347 (18.6%)	**HR 0.82** (0.57 to 1.18)	**3 fewer per 100** (from 8 fewer to 3 more)	⨁◯◯◯ Very low^f,g,h^
**Acute Gastrointestinal Toxicity**											
2	2 Retrospective Study	serious^c^	not serious	not serious	not serious	none	54/389 (13.9%)	134/289 (46.4%)	**OR 6.46** (1.65 to 25.34)	**38 more per 100** (from 12 to 49 more)	⨁⨁⨁◯ Moderate^c^
**Late Gastrointestinal Toxicity**											
3	2 RCTs + 1 Cohort	serious^c^	not serious	not serious	not serious	none	23/407 (5.7%)	85/408 (20.8%)	**OR 4.77** (2.89 to 7.88)	**35 more per 100** (from 22 to 47 more)	⨁⨁⨁◯ Moderate^c^
**Acute Genitourinary Toxicity**											
3	1 RCT + 2 Cohort	serious^i^	not serious	not serious	not serious	none	11/169 (6.5%)	62/286 (21.7%)	**OR 4.79** (1.90 to 12.09)	**35 more per 100** (from 13 to 55 more)	⨁⨁⨁◯ Moderate^i^
**Late Genitourinary Toxicity**											
2	1 RCT + 1 Cohort	serious^c^	not serious	not serious	not serious	none	66/382 (17.3%)	104/383 (27.2%)	**OR 1.84** (1.25 to 2.70)	**14 more per 100** (from 5 to 23 more)	⨁⨁⨁◯ Moderate^c^
**Acute Skin Toxicity**											
2	1 RCT + 1 Cohort	serious^j^	not serious	not serious	not serious	none	70/90 (77.8%)	85/105 (81.0%)	**OR 1.07** (0.31 to 3.68)	**1 more per 100** (from 24 fewer to 13 more)	⨁⨁⨁◯ Moderate^j^
**Hematological Toxicity**											
2	2 Cohort	serious^i^	serious^k^	not serious	not serious	none	14/144 (9.7%)	77/261 (29.5%)	**OR 2.39** (1.18 to 4.85)	**21 more per 100** (from 4 to 37 more)	⨁⨁◯◯ Low^i,k^
**Upper Gastrointestinal Toxicity**											
2	2 Cohort	serious^i^	not serious	not serious	not serious	none	11/144 (7.6%)	108/261 (41.4%)	**OR 7.32** (3.71 to 14.44)	**42 more per 100** (from 31 to 50 more)	⨁⨁⨁◯ Moderate^i^
**Lower Gastrointestinal Toxicity**											
2	2 Cohort	serious^i^	not serious	not serious	not serious	none	18/144 (12.5%)	140/261 (53.6%)	**OR 6.95** (2.38 to 20.25)	**35 more per 100** (from 20 to 42 more)	⨁⨁⨁◯ Moderate^i^

*CI* confidence interval, *HR* hazard ratio, *OR* odds ratio

**Explanations**

^a^Most studies support EBRT + VBT for reducing locoregional recurrence, but variations in radiation techniques exist

^b^Some variations in recurrence rates, but overall findings align with major trials

^c^Sorbe et al. is a well-conducted RCT, while Jin et al. has limitations due to retrospective design

^d^Some studies report differences in distant metastasis rates, while others show no benefit

^e^Distant metastasis rates are generally low, but confidence intervals in studies vary

^f^Some studies are RCTs (Sorbe et al., Sunil et al.), while others are retrospective (Jin et al., Hou et al.)

^g^Studies show mixed results; some suggest no survival benefit with EBRT + VBT

^h^Some studies report slight differences in survival rates, but confidence intervals are wide

^i^observational nature, possible selection bias despite propensity score matching

^j^single-center nature limits generalizability, potential selection bias

^k^differences between centers in patient management may cause variability

## Discussion

The findings regarding the efficacy of adjuvant radiotherapy in the management of endometrial cancer underscore the complexity of treatment outcomes, particularly in relation to overall survival. While the combination of EBRT and VBT has been shown to significantly reduce pelvic recurrence compared to VBT alone, it does not confer a corresponding improvement in overall survival or a reduction in treatment-related toxicity for patients with intermediate to high-risk endometrial cancer. This context sets the stage for further exploration of the therapeutic benefits of EBRT in conjunction with VBT. A notable increase in the application of vaginal brachytherapy was identified throughout both before and after the stratification of risk groups period. Numerous RCTs have indicated that adjuvant radiotherapy contributes to a reduction in local recurrence rates when compared to surgery alone; however, no significant survival advantage has been demonstrated in patients with early-stage endometrial cancer [23–25]. However, two large studies involving patients with stage 1 endometrial cancer demonstrated that the administration of adjuvant radiotherapy, regardless of the radiation modality employed, resulted in improved OS for patients classified as intermediate, high-intermediate, and high risk according to ESMO guidelines [8, 26].

In this study, combination of EBRT and VBT offers superior disease control in patients with intermediate- to high-risk endometrial cancer. These results align with previous studies that suggest EBRT provides better pelvic control by targeting microscopic disease beyond the vaginal cuff, which may not be adequately addressed by VBT alone [5, 9, 17]. The comparative efficacy of EBRT + VBT versus VBT alone in recurrence-free outcomes remains undetermined. Chitapanarux et al. and Wang et al. found no significant difference in LRFS and RRFS, indicating that VBT alone may provide adequate local and regional control [18, 22]. Similarly, DFS outcomes from both studies showed no advantage with EBRT, strengthening the notion that VBT alone may be adequate for disease control. Findings on DMFS were inconsistent, with Chitapanarux et al. favoring VBT alone, while Jin et al. and Sorbe et al. found no difference. RFS results also varied, with Jin et al. reporting a benefit with VBT. IN contrast, Sorbe et al. observed no difference, questioning the added value of EBRT [5, 17]. While EBRT + VBT enhances pelvic control, its impact on recurrence-free survival remains inconclusive, with several studies showing no significant benefit over VBT alone. These results highlight the need for individualized treatment approaches and the importance of further prospective trials to refine optimal radiotherapy strategies in intermediate—to high-risk endometrial cancer.

Despite these benefits in recurrence reduction, the overall survival analysis did not show a statistically significant difference between the two treatment modalities. This finding supports prior research indicating that while EBRT reduces local recurrence, it does not necessarily translate to improved overall survival, likely due to the development of distant metastases, which neither EBRT nor VBT can effectively prevent [20]. Additionally, the PORTEC-2 trial demonstrated that VBT alone provided excellent vaginal control while minimizing gastrointestinal toxicity, reinforcing the argument for individualized treatment approaches [7].

Toxicity remains a critical consideration when determining the optimal radiation strategy. The analysis revealed that EBRT + VBT is associated with a significantly higher risk of both acute and late low-grade gastrointestinal and genitourinary toxicity compared to VBT alone. These adverse effects, particularly gastrointestinal toxicity, may substantially impact quality of life and raise concerns regarding potential overtreatment in certain patient populations. Notably, while EBRT + VBT improves pelvic control, the risk–benefit ratio must be carefully evaluated, particularly for patients with a lower risk of recurrence. The PORTEC study further supports these concerns, demonstrating a significantly higher incidence of late gastrointestinal and urinary complications in patients receiving radiotherapy compared to those managed with observation (26% vs. 4%) [24]. Recent real-world evidence suggests a growing shift toward treatment de-escalation strategies to mitigate toxicity while maintaining oncologic control [27].

Several limitations must be acknowledged. First, the included studies exhibited heterogeneity in patient selection, and follow-up duration, which may influence outcomes. Second, while subgroup analyses attempted to mitigate confounding factors, residual confounding cannot be entirely ruled out. Lastly, the number of randomized controlled trials directly comparing EBRT + VBT with VBT alone in intermediate- to high-risk patients remains limited, highlighting the need for further well-designed prospective trials. Future research should focus on identifying patient subgroups that may derive the most benefit from combined therapy while minimizing unnecessary treatment-related morbidity. Emerging modalities such as intensity-modulated radiotherapy (IMRT) or adaptive radiation therapy may provide avenues to optimize therapeutic efficacy while reducing adverse effects.

## Conclusion

The choice between EBRT + VBT and VBT alone should be individualized based on risk group, patient characteristics, and toxicity considerations. While EBRT provides better pelvic control, it does not improve overall survival and comes with increased toxicity. VBT alone remains a practical option for many intermediate-to high-risk patients, reinforcing the need for risk-adapted treatment strategies.

## Data Availability

No datasets were generated or analysed during the current study.
